# Bis­[4,4′-(propane-1,3-di­yl)­dipiperidin­ium] β-octa­molybdate(VI)

**DOI:** 10.1107/S1600536810013632

**Published:** 2010-04-17

**Authors:** Mohamed Driss, Rekaya Ksiksi, Fatma Ben Amor, Mohamed Faouzi Zid

**Affiliations:** a Laboratoire de Matériaux et Cristallochimie, Faculté des Sciences de Tunis, Université de Tunis El Manar, 2092 Manar II Tunis, Tunisia

## Abstract

The title compound, bis­[4,4′-(propane-1,3-di­yl)­dipiperidin­ium] β-octa­molybdate(VI), (C_13_H_28_N_2_)_2_[Mo_8_O_26_], was produced by hydro­thermal reaction of an acidified aqueous solution of Na_2_MoO_4_·2H_2_O and 4,4′-trimethyl­ene­dipiperidine (*L*). The structure of the title compound consists of β-octa­molybdate(VI) anion clusters and protonated [H_2_ *L*]^2+^ cations. The octa­molybdate anion is located around an inversion center. N—H⋯O hydrogen bonds between the cations and anions ensure the cohesion of the structure and result in a three-dimensional network.

## Related literature

For applications of polyoxometallates (POMs) in catalyst chemistry, see: Pope (1983 ▶). For applications of POMs in materials science, see: Muller *et al.* (1998 ▶). For the introduction of POMs into coordination polymers for the construction of polymers with desired properties, see: Bu *et al.* (2001 ▶); Wu *et al.* (2002 ▶). For the anti­viral and anti­tumour activities of POMs, see: Hasenknopf (2005 ▶); Gerth *et al.* (2005 ▶). For related literature, see: Zebiri *et al.* (2008 ▶); Li & Tan (2008 ▶). For hydrogen-bonding discussion, see: Blessing (1986 ▶); Brown (1976 ▶).
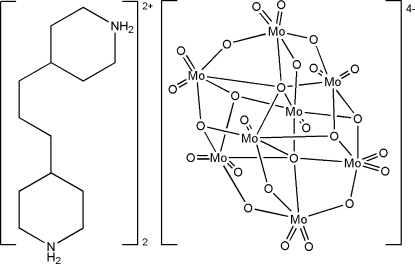


## Experimental

### 

#### Crystal data

(C_13_H_28_N_2_)_2_[Mo_8_O_26_]
*M* *_r_* = 1608.26
Orthorhombic, 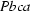

*a* = 23.975 (5) Å
*b* = 13.935 (4) Å
*c* = 13.647 (9) Å
*V* = 4559 (3) Å^3^
*Z* = 4
Mo *K*α radiation
μ = 2.22 mm^−1^
*T* = 298 K
0.4 × 0.3 × 0.2 mm

#### Data collection

Enraf–Nonius CAD-4 diffractometer
Absorption correction: ψ scan (North *et al.*, 1968 ▶) *T*_min_ = 0.556, *T*_max_ = 0.642
5810 measured reflections
4960 independent reflections
3996 reflections with *I* > 2σ(*I*)
*R*_int_ = 0.034
2 standard reflections every 120 min  intensity decay: 4%

#### Refinement

*R*[*F* ^2^ > 2σ(*F* ^2^)] = 0.036
*wR*(*F* ^2^) = 0.099
*S* = 1.08
4960 reflections
289 parameters
H-atom parameters constrained
Δρ_max_ = 1.05 e Å^−3^
Δρ_min_ = −1.27 e Å^−3^

### 

Data collection: *CAD-4 EXPRESS* (Duisenberg, 1992 ▶; Macíček & Yordanov, 1992 ▶); cell refinement: *CAD-4 EXPRESS*; data reduction: *XCAD4* (Harms & Wocadlo, 1995 ▶); program(s) used to solve structure: *SHELXS97* (Sheldrick, 2008 ▶); program(s) used to refine structure: *SHELXL97* (Sheldrick, 2008 ▶); molecular graphics: *ORTEPIII* (Burnett & Johnson, 1996 ▶), *ORTEP-3 for Windows* (Farrugia, 1997 ▶) and *DIAMOND* (Brandenburg, 2001 ▶); software used to prepare material for publication: *WinGX* (Farrugia, 1999 ▶).

## Supplementary Material

Crystal structure: contains datablocks I, global. DOI: 10.1107/S1600536810013632/dn2553sup1.cif

Structure factors: contains datablocks I. DOI: 10.1107/S1600536810013632/dn2553Isup2.hkl

Additional supplementary materials:  crystallographic information; 3D view; checkCIF report

## Figures and Tables

**Table 1:** Hydrogen-bond geometry (Å, °)

*D*—H⋯*A*	*D*—H	H⋯*A*	*D*⋯*A*	*D*—H⋯*A*
N1—H1*B*⋯O5	0.90	2.42	3.312 (6)	172
N2—H2*B*⋯O10^i^	0.90	2.01	2.886 (5)	163

Symmetry code: (i) 
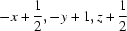
.

**Fig. 1.**
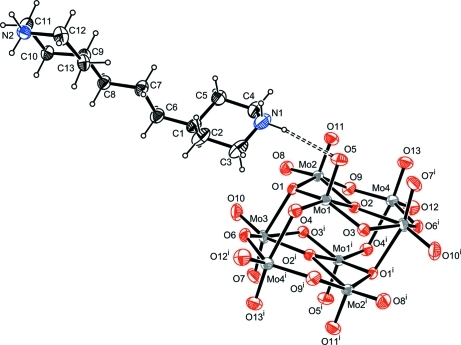
Représentation du polyanion [Mo8O26]4- et d'un cation diprotoné (C13H28N2)2+. Les ellipsoïdes d'agitation thermique ont 30% de probabilité de présence. Les atomes H sont représentés comme des sphères de rayon arbitraire et la liaison H est représentée en trait pointillé. [Code de symétrie: (i) -x + 1, -y + 1, -z]

**Fig. 2.**
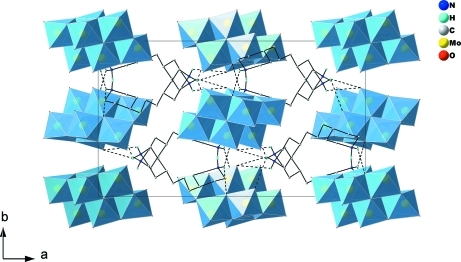
Projection de la structure du composé (C13H28N2)2[Mo8O26] selon c.

## supplementary crystallographic information

### Comment

Les polyoxométallates (POMs) constituent une large famille de clusters d'oxydes métalliques contenant des métaux de transition (principalement V, Mo et W) en leurs degrés d'oxydation les plus élevés (Pope, 1983). La diversité des structures des POMs leur procure une large polyvalence en termes de forme, de polarité, de potentiels redox, de surface, de distribution de charge et d'acidité, ainsi, beaucoup d'applications leur sont possibles dans divers domaines, parmi lesquels, la catalyse, la science des matériaux et chimie des polymères (Pope, 1983; Muller *et al.*, 1998; Bu *et al.*, 2001; Wu *et al.*, 2002). Par ailleurs de récentes études ont montré qu'une gamme de POMs présente des activités antivirales et anti-tumorales (Hasenknopf, 2005; Gerth *et al.*, 2005). Durant notre étude sur ce type de matériaux nous avons isolé une nouvelle phase dont les cristaux sont de qualité et de taille convenables pour une étude par diffraction des rayons X sur monocristal.

L'unité asymétrique du composé (I) consiste en un cation diprotoné 4,4'-triméthylènedipépiridinium et la moitié d'un cluster β-octamolybdate [Mo_8_O_26_]^4-^, chaque cluster étant organisé autour d'un centre d'inversion (Fig. 1). Une liaison hydrogène faible relie un atome d'hydrogène du cation et un atome d'oxygène externe du cluster β-octamolybdate(VI) (Fig. 1).

Des liaisons hydrogène de type N—H···O, satisfaisant la condition NHO supérieur ou égal à 150°, s'établissent entre les cations organiques et les atomes d'oxygène externes des clusters β-octamolybdate(VI), renforçant ainsi la cohésion de la structure générant ainsi une charpente tridimensionnelle (Fig. 2). Ces liaisons sont considérées comme faibles (N···O: 2,885 (6) et 3,312 (7) (Å)), d'après le critère de Brown portant sur les distances et les angles (Brown, 1976; Blessing, 1986). Le composé étudié est comparable à d'autres composés similaires de la littérature, par exemple NH_4_(C_8_H_20_N)_3_[Mo_8_O_26_] (Zebiri *et al.*, 2008) et (C_12_H_20_N_4_)_2_[Mo_8_O_26_] (Li & Tan, 2008). En effet ces deux composés sont constitués de clusters β-octamolybdate discrets et de cations organiques reliés par des liaisons hydrogène de type NH···O. Dans le deuxième exemple cité, bien qu'il n y ait pas partage d'arêtes ni de sommets entre les clusters, l'existence de liaisons hydrogène entre les cations organiques et les clusters confère à la structure le caractère unidimensionnel.

### Experimental

La synthèse a été réalisée par voie hydrothermale avec comme réactifs Na_2_MoO_4_.2H_2_O (0,24 g, 1 mmol) et 4,4'-triméthylènedipépiridine (0,1 g, 1 mmol). Le pH de la solution est ajusté à 4 à l'aide de HCl (6 M). La solution préparée est transvasée dans un récipient en Téflon qui est introduit dans une autoclave en acier. L'ensemble est maintenu sous pression à une température voisine de 150°C pendant deux jours. Le refroidissement jusqu'à température ambiante a été réalisé par paliers de 30° par jour. Des cristaux de forme parallélépipédique, de couleur brune, de taille suffisante et de qualité convenable pour une étude structurale sont obtenus.

### Refinement

All H atoms have been positioned geometrically using AFIX23 and AFIX13 instructions of SHELXL97 (Sheldrick, 2008) with the constraint U_iso_(H) = 1.2U_eq_(C).

### Crystal data

**Table:**

(C_13_H_28_N_2_)_2_[Mo_8_O_26_]	*F*(000) = 3136
*M**_r_* = 1608.26	*D*_x_ = 2.343 Mg m^−^^3^
Orthorhombic, *P**b**c**a*	Mo *K*α radiation, λ = 0.71073 Å
Hall symbol: -P 2ac 2ab	Cell parameters from 25 reflections
*a* = 23.975 (5) Å	θ = 12–15°
*b* = 13.935 (4) Å	µ = 2.22 mm^−^^1^
*c* = 13.647 (9) Å	*T* = 298 K
*V* = 4559 (3) Å^3^	Prism, brown
*Z* = 4	0.4 × 0.3 × 0.2 mm

### Data collection

**Table:**

Enraf–Nonius CAD-4 diffractometer	3996 reflections with *I* > 2σ(*I*)
Radiation source: fine-focus sealed tube	*R*_int_ = 0.034
graphite	θ_max_ = 27.0°, θ_min_ = 2.2°
ω/2θ scans	*h* = −30→0
Absorption correction: ψ scan (North *et al.*, 1968)	*k* = −1→17
*T*_min_ = 0.556, *T*_max_ = 0.642	*l* = −1→17
5810 measured reflections	2 standard reflections every 120 min
4960 independent reflections	intensity decay: 4%

### Refinement

**Table:**

Refinement on *F*^2^	Primary atom site location: structure-invariant direct methods
Least-squares matrix: full	Secondary atom site location: difference Fourier map
*R*[*F*^2^ > 2σ(*F*^2^)] = 0.036	Hydrogen site location: inferred from neighbouring sites
*wR*(*F*^2^) = 0.099	H-atom parameters constrained
*S* = 1.08	*w* = 1/[σ^2^(*F*_o_^2^) + (0.050*P*)^2^ + 9.1911*P*] where *P* = (*F*_o_^2^ + 2*F*_c_^2^)/3
4960 reflections	(Δ/σ)_max_ = 0.001
289 parameters	Δρ_max_ = 1.05 e Å^−^^3^
0 restraints	Δρ_min_ = −1.27 e Å^−^^3^

### Special details

**Table:**

Geometry. All esds (except the esd in the dihedral angle between two l.s. planes) are estimated using the full covariance matrix. The cell esds are taken into account individually in the estimation of esds in distances, angles and torsion angles; correlations between esds in cell parameters are only used when they are defined by crystal symmetry. An approximate (isotropic) treatment of cell esds is used for estimating esds involving l.s. planes.
Refinement. Refinement of *F*^2^ against ALL reflections. The weighted *R*-factor wR and goodness of fit *S* are based on *F*^2^, conventional *R*-factors *R* are based on *F*, with *F* set to zero for negative *F*^2^. The threshold expression of *F*^2^ > σ(*F*^2^) is used only for calculating *R*-factors(gt) etc. and is not relevant to the choice of reflections for refinement. *R*-factors based on *F*^2^ are statistically about twice as large as those based on *F*, and *R*- factors based on ALL data will be even larger.

### Fractional atomic coordinates and isotropic or equivalent isotropic displacement parameters (Å^2^)

**Table:**

	*x*	*y*	*z*	*U*_iso_*/*U*_eq_	
N1	0.4488 (2)	0.3151 (4)	0.3293 (4)	0.0540 (15)	
H1A	0.4463	0.2575	0.3590	0.065*	
H1B	0.4708	0.3081	0.2764	0.065*	
N2	0.15957 (16)	0.2275 (3)	0.8306 (3)	0.0294 (9)	
H2A	0.1468	0.1813	0.8708	0.035*	
H2B	0.1300	0.2547	0.8008	0.035*	
C1	0.3795 (2)	0.4370 (4)	0.4548 (4)	0.0312 (11)	
H1	0.3854	0.4959	0.4170	0.037*	
C2	0.3548 (3)	0.3633 (5)	0.3856 (4)	0.0449 (15)	
H20	0.3187	0.3857	0.3631	0.054*	
H21	0.3490	0.3036	0.4208	0.054*	
C3	0.3924 (3)	0.3444 (5)	0.2965 (4)	0.0463 (15)	
H31	0.3762	0.2941	0.2564	0.056*	
H32	0.3950	0.4021	0.2571	0.056*	
C4	0.4753 (3)	0.3838 (5)	0.3975 (4)	0.0504 (16)	
H41	0.4819	0.4442	0.3641	0.060*	
H42	0.5109	0.3587	0.4192	0.060*	
C5	0.4370 (2)	0.4003 (5)	0.4866 (4)	0.0401 (13)	
H51	0.4327	0.3406	0.5223	0.048*	
H52	0.4542	0.4468	0.5302	0.048*	
C6	0.3415 (2)	0.4628 (4)	0.5419 (4)	0.0352 (12)	
H61	0.3615	0.5075	0.5835	0.042*	
H62	0.3091	0.4961	0.5165	0.042*	
C7	0.3214 (2)	0.3803 (4)	0.6050 (4)	0.0340 (12)	
H71	0.3532	0.3498	0.6359	0.041*	
H72	0.3032	0.3330	0.5638	0.041*	
C8	0.2805 (2)	0.4140 (4)	0.6843 (4)	0.0295 (10)	
H81	0.2501	0.4479	0.6526	0.035*	
H82	0.2997	0.4596	0.7262	0.035*	
C9	0.25557 (19)	0.3348 (3)	0.7494 (3)	0.0261 (9)	
H9	0.2860	0.3038	0.7853	0.031*	
C10	0.2149 (2)	0.3782 (3)	0.8239 (3)	0.0274 (10)	
H101	0.2345	0.4251	0.8637	0.033*	
H102	0.1853	0.4112	0.7893	0.033*	
C11	0.1898 (2)	0.3022 (4)	0.8900 (4)	0.0338 (11)	
H111	0.2191	0.2718	0.9279	0.041*	
H112	0.1639	0.3321	0.9354	0.041*	
C12	0.1969 (2)	0.1832 (4)	0.7545 (4)	0.0365 (12)	
H121	0.1751	0.1405	0.7135	0.044*	
H122	0.2254	0.1453	0.7868	0.044*	
C13	0.22476 (19)	0.2581 (4)	0.6904 (4)	0.0299 (10)	
H131	0.1966	0.2889	0.6501	0.036*	
H132	0.2510	0.2265	0.6470	0.036*	
Mo1	0.484896 (16)	0.38831 (3)	0.05948 (3)	0.02043 (11)	
Mo2	0.555686 (16)	0.54337 (3)	0.18157 (3)	0.02254 (11)	
Mo3	0.422788 (16)	0.61244 (3)	0.11200 (3)	0.02489 (11)	
Mo4	0.650139 (17)	0.54832 (3)	0.00726 (3)	0.02559 (11)	
O1	0.47977 (13)	0.4851 (2)	0.1631 (2)	0.0225 (6)	
O2	0.55436 (13)	0.4788 (2)	0.0253 (2)	0.0211 (6)	
O3	0.49781 (13)	0.3539 (2)	−0.0778 (2)	0.0230 (6)	
O4	0.41461 (13)	0.3525 (3)	0.0625 (2)	0.0273 (7)	
O5	0.51905 (15)	0.3005 (3)	0.1209 (3)	0.0347 (8)	
O6	0.36637 (13)	0.5174 (3)	0.1144 (2)	0.0279 (7)	
O7	0.39269 (15)	0.7001 (3)	0.0433 (3)	0.0387 (9)	
O8	0.54174 (16)	0.6221 (3)	0.2737 (3)	0.0353 (8)	
O9	0.61609 (14)	0.6091 (3)	0.1208 (2)	0.0295 (8)	
O10	0.42016 (15)	0.6529 (3)	0.2296 (3)	0.0420 (10)	
O11	0.58676 (15)	0.4471 (3)	0.2357 (3)	0.0336 (8)	
O12	0.70309 (16)	0.6243 (3)	−0.0214 (3)	0.0415 (10)	
O13	0.68141 (15)	0.4542 (3)	0.0665 (3)	0.0366 (9)	

### Atomic displacement parameters (Å^2^)

**Table:**

	*U*^11^	*U*^22^	*U*^33^	*U*^12^	*U*^13^	*U*^23^
N1	0.089 (4)	0.040 (3)	0.033 (3)	0.024 (3)	0.017 (3)	0.007 (2)
N2	0.0215 (18)	0.028 (2)	0.038 (2)	−0.0026 (17)	−0.0008 (17)	0.0090 (18)
C1	0.040 (3)	0.027 (3)	0.027 (2)	−0.005 (2)	0.004 (2)	0.003 (2)
C2	0.055 (4)	0.051 (3)	0.029 (3)	−0.020 (3)	0.004 (2)	0.003 (3)
C3	0.066 (4)	0.047 (3)	0.026 (3)	−0.020 (3)	0.005 (3)	−0.003 (3)
C4	0.045 (3)	0.071 (5)	0.035 (3)	0.007 (3)	0.006 (3)	0.004 (3)
C5	0.038 (3)	0.050 (3)	0.033 (3)	0.000 (3)	0.003 (2)	0.003 (3)
C6	0.041 (3)	0.031 (3)	0.034 (3)	0.000 (2)	0.009 (2)	0.006 (2)
C7	0.035 (3)	0.032 (3)	0.034 (3)	−0.003 (2)	0.010 (2)	0.002 (2)
C8	0.032 (3)	0.027 (2)	0.029 (2)	0.000 (2)	0.005 (2)	0.001 (2)
C9	0.023 (2)	0.027 (2)	0.028 (2)	−0.0002 (19)	0.0011 (18)	0.002 (2)
C10	0.030 (2)	0.025 (2)	0.028 (2)	−0.0036 (19)	0.0047 (19)	−0.0025 (19)
C11	0.034 (3)	0.042 (3)	0.026 (2)	−0.004 (2)	0.005 (2)	0.001 (2)
C12	0.033 (3)	0.026 (2)	0.051 (3)	−0.002 (2)	0.005 (2)	−0.007 (2)
C13	0.023 (2)	0.030 (2)	0.036 (2)	−0.0020 (19)	0.005 (2)	−0.004 (2)
Mo1	0.0216 (2)	0.0201 (2)	0.01959 (19)	−0.00003 (14)	−0.00049 (14)	0.00314 (15)
Mo2	0.0213 (2)	0.0275 (2)	0.01882 (19)	−0.00030 (15)	−0.00195 (14)	0.00103 (15)
Mo3	0.0206 (2)	0.0281 (2)	0.0260 (2)	0.00483 (16)	0.00207 (15)	−0.00384 (16)
Mo4	0.0195 (2)	0.0321 (2)	0.0252 (2)	−0.00429 (16)	−0.00038 (15)	0.00520 (17)
O1	0.0205 (15)	0.0300 (17)	0.0170 (14)	0.0002 (13)	0.0031 (12)	0.0006 (13)
O2	0.0197 (15)	0.0237 (16)	0.0198 (15)	−0.0001 (12)	−0.0010 (12)	0.0010 (12)
O3	0.0209 (15)	0.0241 (16)	0.0240 (15)	−0.0016 (13)	−0.0005 (13)	−0.0003 (13)
O4	0.0245 (16)	0.0300 (18)	0.0272 (17)	−0.0058 (14)	0.0000 (13)	0.0027 (14)
O5	0.037 (2)	0.0296 (19)	0.0371 (19)	0.0021 (16)	−0.0061 (16)	0.0099 (16)
O6	0.0201 (16)	0.0387 (19)	0.0249 (16)	0.0002 (14)	0.0026 (13)	−0.0009 (15)
O7	0.0318 (19)	0.037 (2)	0.048 (2)	0.0123 (17)	−0.0005 (17)	0.0045 (18)
O8	0.036 (2)	0.042 (2)	0.0280 (17)	0.0018 (17)	−0.0007 (15)	−0.0062 (15)
O9	0.0248 (17)	0.037 (2)	0.0266 (16)	−0.0101 (14)	−0.0020 (14)	0.0012 (15)
O10	0.0317 (19)	0.059 (3)	0.035 (2)	0.0075 (19)	0.0045 (16)	−0.0158 (19)
O11	0.0270 (18)	0.039 (2)	0.0353 (19)	0.0033 (16)	−0.0050 (15)	0.0038 (16)
O12	0.0287 (19)	0.053 (2)	0.043 (2)	−0.0137 (18)	−0.0003 (17)	0.0127 (19)
O13	0.0295 (19)	0.044 (2)	0.036 (2)	0.0029 (17)	−0.0057 (16)	0.0088 (17)

### Geometric parameters (Å, °)

**Table:**

N1—C4	1.478 (9)	C10—H101	0.9700
N1—C3	1.482 (9)	C10—H102	0.9700
N1—H1A	0.9000	C11—H111	0.9700
N1—H1B	0.9000	C11—H112	0.9700
N2—C12	1.503 (6)	C12—C13	1.517 (7)
N2—C11	1.506 (6)	C12—H121	0.9700
N2—H2A	0.9000	C12—H122	0.9700
N2—H2B	0.9000	C13—H131	0.9700
C1—C2	1.516 (8)	C13—H132	0.9700
C1—C5	1.534 (8)	Mo1—O5	1.695 (3)
C1—C6	1.540 (7)	Mo1—O4	1.758 (3)
C1—H1	0.9800	Mo1—O1	1.958 (3)
C2—C3	1.536 (8)	Mo1—O3	1.958 (3)
C2—H20	0.9700	Mo1—O2	2.140 (3)
C2—H21	0.9700	Mo1—O2^i^	2.378 (3)
C3—H31	0.9700	Mo1—Mo2	3.213 (2)
C3—H32	0.9700	Mo2—O8	1.702 (4)
C4—C5	1.540 (8)	Mo2—O11	1.703 (4)
C4—H41	0.9700	Mo2—O9	1.903 (3)
C4—H42	0.9700	Mo2—O1	2.009 (3)
C5—H51	0.9700	Mo2—O2	2.316 (3)
C5—H52	0.9700	Mo2—O3^i^	2.387 (3)
C6—C7	1.514 (7)	Mo3—O7	1.700 (4)
C6—H61	0.9700	Mo3—O10	1.703 (4)
C6—H62	0.9700	Mo3—O6	1.894 (3)
C7—C8	1.534 (7)	Mo3—O3^i^	2.015 (3)
C7—H71	0.9700	Mo3—O2^i^	2.329 (3)
C7—H72	0.9700	Mo3—O1	2.346 (3)
C8—C9	1.538 (7)	Mo4—O12	1.699 (4)
C8—H81	0.9700	Mo4—O13	1.713 (4)
C8—H82	0.9700	Mo4—O6^i^	1.937 (3)
C9—C13	1.528 (7)	Mo4—O9	1.945 (4)
C9—C10	1.533 (6)	Mo4—O4^i^	2.286 (3)
C9—H9	0.9800	Mo4—O2	2.505 (3)
C10—C11	1.516 (7)		
			
C4—N1—C3	113.8 (5)	O5—Mo1—O1	99.82 (16)
C4—N1—H1A	108.8	O4—Mo1—O1	96.81 (15)
C3—N1—H1A	108.8	O5—Mo1—O3	102.72 (16)
C4—N1—H1B	108.8	O4—Mo1—O3	95.99 (14)
C3—N1—H1B	108.8	O1—Mo1—O3	150.38 (13)
H1A—N1—H1B	107.7	O5—Mo1—O2	99.06 (15)
C12—N2—C11	111.7 (4)	O4—Mo1—O2	156.65 (14)
C12—N2—H2A	109.3	O1—Mo1—O2	78.50 (12)
C11—N2—H2A	109.3	O3—Mo1—O2	79.18 (12)
C12—N2—H2B	109.3	O5—Mo1—O2^i^	174.01 (15)
C11—N2—H2B	109.3	O4—Mo1—O2^i^	81.53 (13)
H2A—N2—H2B	107.9	O1—Mo1—O2^i^	77.87 (12)
C2—C1—C5	107.6 (5)	O3—Mo1—O2^i^	77.75 (12)
C2—C1—C6	114.0 (5)	O2—Mo1—O2^i^	75.12 (12)
C5—C1—C6	113.0 (4)	O5—Mo1—Mo2	88.51 (13)
C2—C1—H1	107.3	O4—Mo1—Mo2	133.24 (11)
C5—C1—H1	107.3	O1—Mo1—Mo2	36.43 (9)
C6—C1—H1	107.3	O3—Mo1—Mo2	125.24 (9)
C1—C2—C3	112.3 (5)	O2—Mo1—Mo2	46.06 (8)
C1—C2—H20	109.1	O2^i^—Mo1—Mo2	86.41 (8)
C3—C2—H20	109.1	O8—Mo2—O11	105.86 (18)
C1—C2—H21	109.1	O8—Mo2—O9	99.28 (17)
C3—C2—H21	109.1	O11—Mo2—O9	103.59 (17)
H20—C2—H21	107.9	O8—Mo2—O1	100.10 (16)
N1—C3—C2	110.1 (5)	O11—Mo2—O1	97.61 (15)
N1—C3—H31	109.6	O9—Mo2—O1	145.99 (13)
C2—C3—H31	109.6	O8—Mo2—O2	158.21 (15)
N1—C3—H32	109.6	O11—Mo2—O2	95.70 (15)
C2—C3—H32	109.6	O9—Mo2—O2	78.23 (13)
H31—C3—H32	108.2	O1—Mo2—O2	73.42 (12)
N1—C4—C5	109.8 (5)	O8—Mo2—O3^i^	86.92 (15)
N1—C4—H41	109.7	O11—Mo2—O3^i^	164.71 (15)
C5—C4—H41	109.7	O9—Mo2—O3^i^	82.07 (13)
N1—C4—H42	109.7	O1—Mo2—O3^i^	71.40 (12)
C5—C4—H42	109.7	O2—Mo2—O3^i^	71.29 (11)
H41—C4—H42	108.2	O8—Mo2—Mo1	135.47 (13)
C1—C5—C4	111.2 (5)	O11—Mo2—Mo1	85.78 (13)
C1—C5—H51	109.4	O9—Mo2—Mo1	119.95 (11)
C4—C5—H51	109.4	O1—Mo2—Mo1	35.37 (9)
C1—C5—H52	109.4	O2—Mo2—Mo1	41.72 (8)
C4—C5—H52	109.4	O3^i^—Mo2—Mo1	79.14 (8)
H51—C5—H52	108.0	O7—Mo3—O10	105.4 (2)
C7—C6—C1	116.8 (4)	O7—Mo3—O6	102.07 (17)
C7—C6—H61	108.1	O10—Mo3—O6	100.89 (18)
C1—C6—H61	108.1	O7—Mo3—O3^i^	96.11 (16)
C7—C6—H62	108.1	O10—Mo3—O3^i^	100.15 (16)
C1—C6—H62	108.1	O6—Mo3—O3^i^	147.28 (14)
H61—C6—H62	107.3	O7—Mo3—O2^i^	92.78 (16)
C6—C7—C8	111.9 (4)	O10—Mo3—O2^i^	161.40 (16)
C6—C7—H71	109.2	O6—Mo3—O2^i^	78.48 (13)
C8—C7—H71	109.2	O3^i^—Mo3—O2^i^	73.64 (12)
C6—C7—H72	109.2	O7—Mo3—O1	162.66 (15)
C8—C7—H72	109.2	O10—Mo3—O1	89.54 (16)
H71—C7—H72	107.9	O6—Mo3—O1	83.19 (13)
C7—C8—C9	115.9 (4)	O3^i^—Mo3—O1	72.21 (12)
C7—C8—H81	108.3	O2^i^—Mo3—O1	71.90 (11)
C9—C8—H81	108.3	O12—Mo4—O13	104.98 (19)
C7—C8—H82	108.3	O12—Mo4—O6^i^	104.46 (17)
C9—C8—H82	108.3	O13—Mo4—O6^i^	97.60 (17)
H81—C8—H82	107.4	O12—Mo4—O9	103.06 (18)
C13—C9—C10	108.6 (4)	O13—Mo4—O9	98.10 (17)
C13—C9—C8	112.7 (4)	O6^i^—Mo4—O9	143.39 (14)
C10—C9—C8	110.3 (4)	O12—Mo4—O4^i^	92.01 (16)
C13—C9—H9	108.4	O13—Mo4—O4^i^	163.01 (15)
C10—C9—H9	108.4	O6^i^—Mo4—O4^i^	77.87 (14)
C8—C9—H9	108.4	O9—Mo4—O4^i^	77.50 (14)
C11—C10—C9	111.8 (4)	O12—Mo4—O2	161.62 (16)
C11—C10—H101	109.2	O13—Mo4—O2	93.37 (15)
C9—C10—H101	109.2	O6^i^—Mo4—O2	73.37 (12)
C11—C10—H102	109.2	O9—Mo4—O2	72.87 (12)
C9—C10—H102	109.2	O4^i^—Mo4—O2	69.64 (11)
H101—C10—H102	107.9	Mo1—O1—Mo2	108.20 (14)
N2—C11—C10	110.7 (4)	Mo1—O1—Mo3	110.06 (14)
N2—C11—H111	109.5	Mo2—O1—Mo3	105.02 (14)
C10—C11—H111	109.5	Mo1—O2—Mo2	92.22 (11)
N2—C11—H112	109.5	Mo1—O2—Mo3^i^	92.13 (12)
C10—C11—H112	109.5	Mo2—O2—Mo3^i^	161.78 (15)
H111—C11—H112	108.1	Mo1—O2—Mo1^i^	104.88 (12)
N2—C12—C13	112.2 (4)	Mo2—O2—Mo1^i^	98.66 (12)
N2—C12—H121	109.2	Mo3^i^—O2—Mo1^i^	97.29 (12)
C13—C12—H121	109.2	Mo1—O2—Mo4	164.32 (15)
N2—C12—H122	109.2	Mo2—O2—Mo4	85.83 (10)
C13—C12—H122	109.2	Mo3^i^—O2—Mo4	85.22 (10)
H121—C12—H122	107.9	Mo1^i^—O2—Mo4	90.78 (11)
C12—C13—C9	112.9 (4)	Mo1—O3—Mo3^i^	108.30 (14)
C12—C13—H131	109.0	Mo1—O3—Mo2^i^	109.63 (14)
C9—C13—H131	109.0	Mo3^i^—O3—Mo2^i^	103.34 (13)
C12—C13—H132	109.0	Mo1—O4—Mo4^i^	118.04 (17)
C9—C13—H132	109.0	Mo3—O6—Mo4^i^	117.50 (17)
H131—C13—H132	107.8	Mo2—O9—Mo4	117.21 (17)
O5—Mo1—O4	104.29 (17)		

Symmetry codes: (i) −*x*+1, −*y*+1, −*z*.

### Hydrogen-bond geometry (Å, °)

**Table:**

*D*—H···*A*	*D*—H	H···*A*	*D*···*A*	*D*—H···*A*
N1—H1B···O5	0.90	2.42	3.312 (6)	172
N2—H2B···O10^ii^	0.90	2.01	2.886 (5)	163

Symmetry codes: (ii) −*x*+1/2, −*y*+1, *z*+1/2.
